# Notch1 is a prognostic factor that is distinctly activated in the classical and proneural subtype of glioblastoma and that promotes glioma cell survival via the NF-κB(p65) pathway

**DOI:** 10.1038/s41419-017-0119-z

**Published:** 2018-02-06

**Authors:** Long Hai, Chen Zhang, Tao Li, Xingchen Zhou, Bo Liu, Shuai Li, Meng Zhu, Yu Lin, Shengping Yu, Kai Zhang, Bingcheng Ren, Haolang Ming, Yubao Huang, Lei Chen, Pengfei Zhao, Hua Zhou, Tao Jiang, Xuejun Yang

**Affiliations:** 1 Department of Neurosurgery, Tianjin Medical University General Hospital, Tianjin, 300052 China; 2 Laboratory of Neuro-Oncology, Tianjin Neurological Institute, Tianjin, 300052 China; 3 Key Laboratory of Post-Trauma Neuro-Repair and Regeneration in Central Nervous System, Ministry of Education, Tianjin, 300052 China; 4 Tianjin Key Laboratory of Injuries, Variations and Regeneration of Nervous System, Tianjin, 300052 China; 5 Chinese Glioma Cooperative Group (CGCG), 6 Tiantanxi Li, Beijing, 100050 China; 6 Department of Neuro-Oncology, The University of Texas MD Anderson Cancer Center, Houston, TX 77030 USA; 7 Department of Neurosurgery, The Affliated Hospital of Qingdao University, Qingdao, Shandong 266003 China; 8 Department of Neurosurgery, Beijing Tiantan Hospital, Capital Medical University, Beijing, 100050 China

## Abstract

Glioblastomas (GBMs) are the most prevalent and devastating primary intracranial malignancies and have extensive heterogeneity. Notch1 signaling is a more complex process in the development of numerous cell and tissue types, including gliomagenesis and progression, and is upregulated in glioma-initiating cells. However, the contradictory expression of Notch1 among lower grade gliomas and GBMs confounds our understanding of GBM biology and has made identifying effective therapies difficult. In this study, we validated that Notch1 and NF-κB(p65) are highly expressed in the classical and proneural subtypes of GBM using the data set from The Cancer Genome Atlas (TCGA) and the Chinese Glioma Genome Atlas (CGGA). DAPT and shRNA targeting Notch1 decreased NF-κB(p65) expression, suppressed cell proliferation, and induced apoptosis of GBM cells in vitro and in vivo. Furthermore, we illustrated that the intracellular Notch could bind with NF-κB(p65) in GBM cells. These findings suggest that the cross-talk between Notch1 signaling and NF-κB(p65) could contribute to the proliferation and apoptosis of glioma, and this discovery could help drive the design of more effective therapies in Notch1-targeted clinical trials.

## Introduction

Glioblastomas (GBMs) are the most prevalent and devastating primary intracranial malignancies and are characterized by extensive heterogeneity at cellular and molecular levels^1^. Despite improvements in the current standards of care, patients who suffer from GBM have a median survival time of only 14.6 months^2^. As refractory tumors in humans, GBMs were the one of the first cancers profiled by The Cancer Genome Atlas (TCGA) project^3^. Based on genomic abnormalities and gene expression, TCGA described four molecular subtypes of GBM known as classical, mesenchymal, neural, and proneural, which provided a basis for understanding the inherent heterogeneity of GBMs^4^.

Cancer stem cell models have been proposed to explain the origin and maintenance of tumor heterogeneity^5^. In GBMs, glioma stem cells (GSCs) or glioma-initiating cells (GICs) were identified more than a decade ago, which are also inherently responsible for the tumor growth, therapeutic resistance, and tumor relapse^6^. Notch signaling, an evolutionarily conserved pathway that mediates direct cell–cell interactions, has been shown to regulate neural stem cells (NSCs) and GSCs during normal neurogenesis and pathological carcinogenesis, respectively. Our previous study focused on how Notch1 signaling maintained the stem cell phenotype in GBMs^7^. As is commonly known, four Notch receptors (Notch1–4) and five Notch ligands including Jagged-1 and 2 and Delta-like-1, 3, and 4 have been identified in mammals^8^. Binding of a Jagged or Delta-like ligand on one cell to Notch on an adjacent cell triggers enzymatic cleavages that liberate the Notch intracellular domain (NICD). The NICD travels to the nucleus, where it interacts with the DNA-binding protein RBP-Jκ, activates transcription via a CSL (CBF1/RBP-Jκ/Suppressor of Hairless/LAG-1) transcription factor and triggers a cascade of events leading to the upregulation of the Hes and Hey families^9,10^.

Recently, several studies have reported the expression features of Notch1 in gliomas with different results regarding tumor progression and prognosis^11–14^. The discrepancies of Notch1 expression in GBMs caught our attention. Espinoza et al. reported that Notch1 was abnormally expressed in gliomas of all grades but was absent in a subset of grade IV gliomas^12^. In contrast, some published data identified Notch1 as overexpressed in GBMs^11,13,14^. These inconsistent profiles of Notch1 expression reported by different studies perhaps reflect the extensive heterogeneity of GBMs. Additionally, at least, these variations could be partly attributed to the failure of Notch1-targeted clinical trials for GBMs. In this article, we validated Notch1 expression in GBMs on four gene expression profiling cohorts of gliomas.

Notch1 has been reported to cross-talk with various pathways involved in growth and apoptosis, including interactions with NF-κB(Nuclear factor-κB). The NF-κB transcription factor family consists of NF-κB1(p50), NF-κB2(p52), RelA(p65), RelB, and cRel, all of which can form different heterodimers or homodimers^15^. Under most circumstances, NF-κB/Rel dimers are sequestered in the cytoplasm by a member of the IκB(Inhibitor-κB) family of inhibitory proteins. In general, various stimuli can promote the dissociation of the inactive NF-κB/IκB complexes via IKK (IκB kinase) activation, which results in the serine phosphorylation and degradation of IκB, and the consequent translocation of NF-κB/Rel dimers into the nucleus^16^. Once translocated to the nucleus, the NF-κB dimers can bind to DNA and regulate the transcription of various genes involved in several aspects of cellular activities. Some downstream target genes of NF-κB are Bcl-2 (the inhibitor of apoptosis proteins) and cyclin D1 (facilitating tumor survival and proliferation)^17^. Specifically, Notch1 has been reported to induce NF-κB2(p52) promoter activity via RBP-Jκ and induce expression of several NF-κB subunits^18,19^. Other investigators have shown that NF-κB(p65) can activate the Notch1 signaling pathway by binding to the Notch1 promoter^20^.

However, little is known about the expression of Notch1 and NF-κB(p65) in the different GBM subtypes and how Notch1 regulates the NF-κB(p65) signaling pathways in GBM. In this study, we assessed the association between Notch1 and NF-κB(p65) expression in GBM samples. Moreover, we first showed that Notch1 promoted GBM development through NICD binding with NF-κB(p65), which affected proliferation and apoptosis in vitro and vivo. Therefore, combined targeting of Notch1 signaling and the NF-κB(p65) pathways may be a novel therapeutic intervention for treating GBM patients.

## Results

### Notch1 expression was increased in GBM and correlates with RELA (NF-κB(p65)) expression

**Fig. 1:**
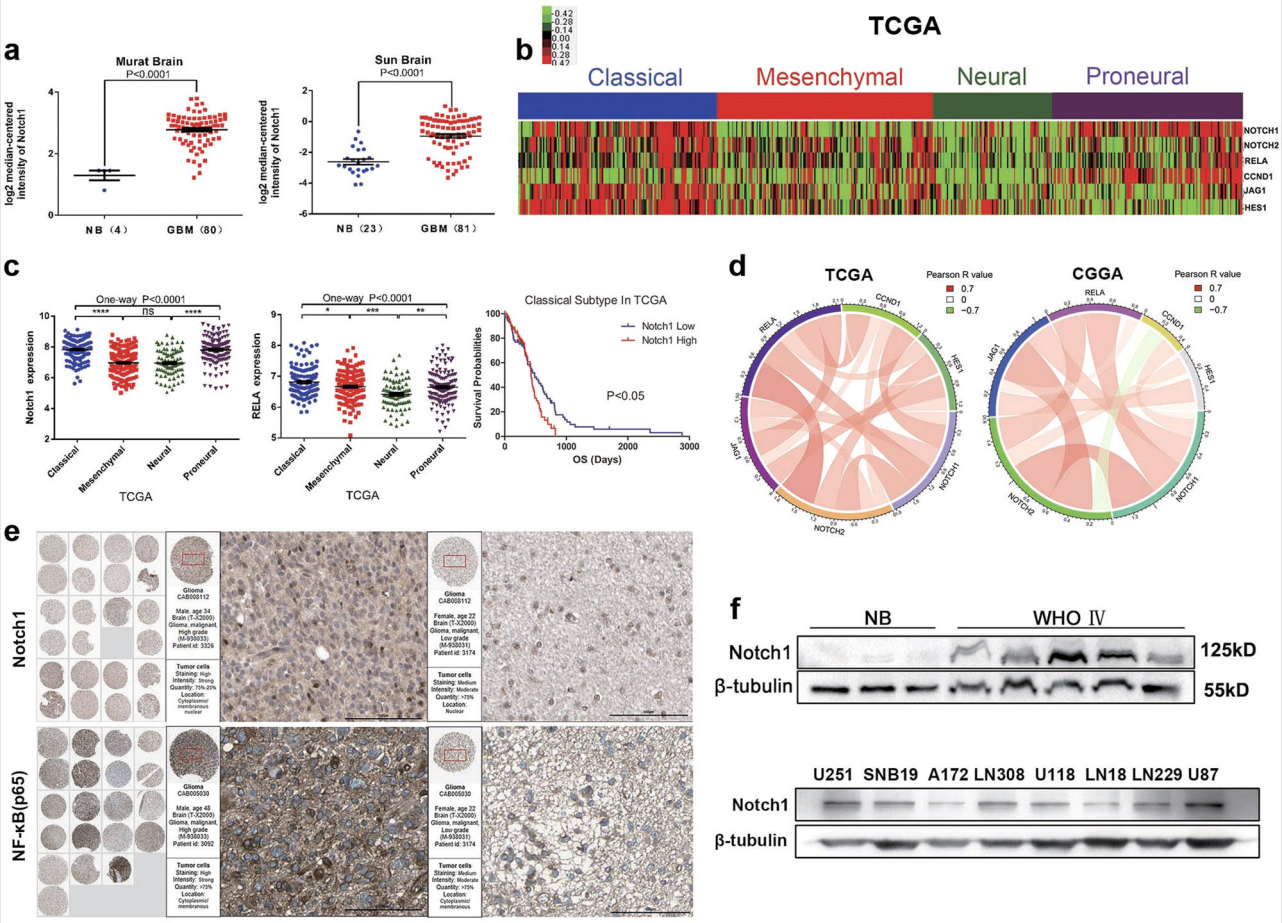
Notch1 expression was increased in GBM, and elevated Notch1 expression was a prognostic indicator of poor survival in patients with classical GBM **a** Notch1 expression was analyzed in GBM tissues and non-tumor brain tissues from the Murat Brain and Sun Brain data sets. NB, non-tumor brain tissue. **b**, **c** Notch1 mRNA expression was analyzed in GBM tissues from the TCGA data sets. Kaplan–Meier survival curve analysis indicated that patients with Notch1 overexpression had a significantly shorter overall survival in the classical subtype of GBM. **d** Pearson correlation analysis between the Notch1 pathway and NF-κB(p65) (RELA) in TCGA and CGGA data sets. **e** Notch1 and NF-κB(p65) protein expression levels were elevated in primary glioma patient samples as indicated by the Human Protein Atlas database (http://www.proteinatlas.org/). **f** The levels of Notch1 in GBM tumor tissues and glioma cell lines were detected by western blotting

We first analyzed Notch1 mRNA expression in Murat Brain and Sun Brain data sets from Oncomine. The mRNA expression and WB (Western Blotting) results showed that Notch1 was overexpressed in GBM samples compared with normal brain controls (Figs. 1a, f). We then examined the mRNA microarray data from TCGA (Figs. 1b, c) and the Chinese Glioma Genome Atlas (CGGA; Supplementary Figures S1b, d and e). The results of the cluster analysis revealed that the Notch1 signaling pathway and RELA (NF-κB(p65)) were significantly upregulated in classical and proneural subtypes of GBM. Next, we evaluated the prognostic values of Notch1 by Kaplan–Meier survival curve analysis in classical GBM. Patients with higher Notch1 expression had a shorter overall survival (Fig. 1c). Furthermore, based on the Pearson correlation analysis of TCGA Pan-Cancer (Supplementary Table S4), Notch1 expression was positively correlated with RELA (NF-κB(p65)) expression in GBM. We additionally performed a correlation analysis in TCGA and CGGA, which also showed a positive correlation between Notch1 and RELA (Fig. 1d). The PPI (Protein-protein interaction) network and immunohistochemical analysis also confirmed this finding (Supplementary Figures S1a and g). The immunofluorescence results indicated that Notch1 and NF-κB(p65) were colocalized in the same cells within the GBM tissue (Supplementary Figure S1 h).

### CD133+ glioma neurospheres exhibited high Notch1 activity

Several groups demonstrated that GBMs contain self-renewing GICs, which are resistant to radiation and chemotherapy^21^. To confirm that GICs harbored elevated Notch1 activity, we established glioma neurospheres in vitro.

**Fig. 2:**
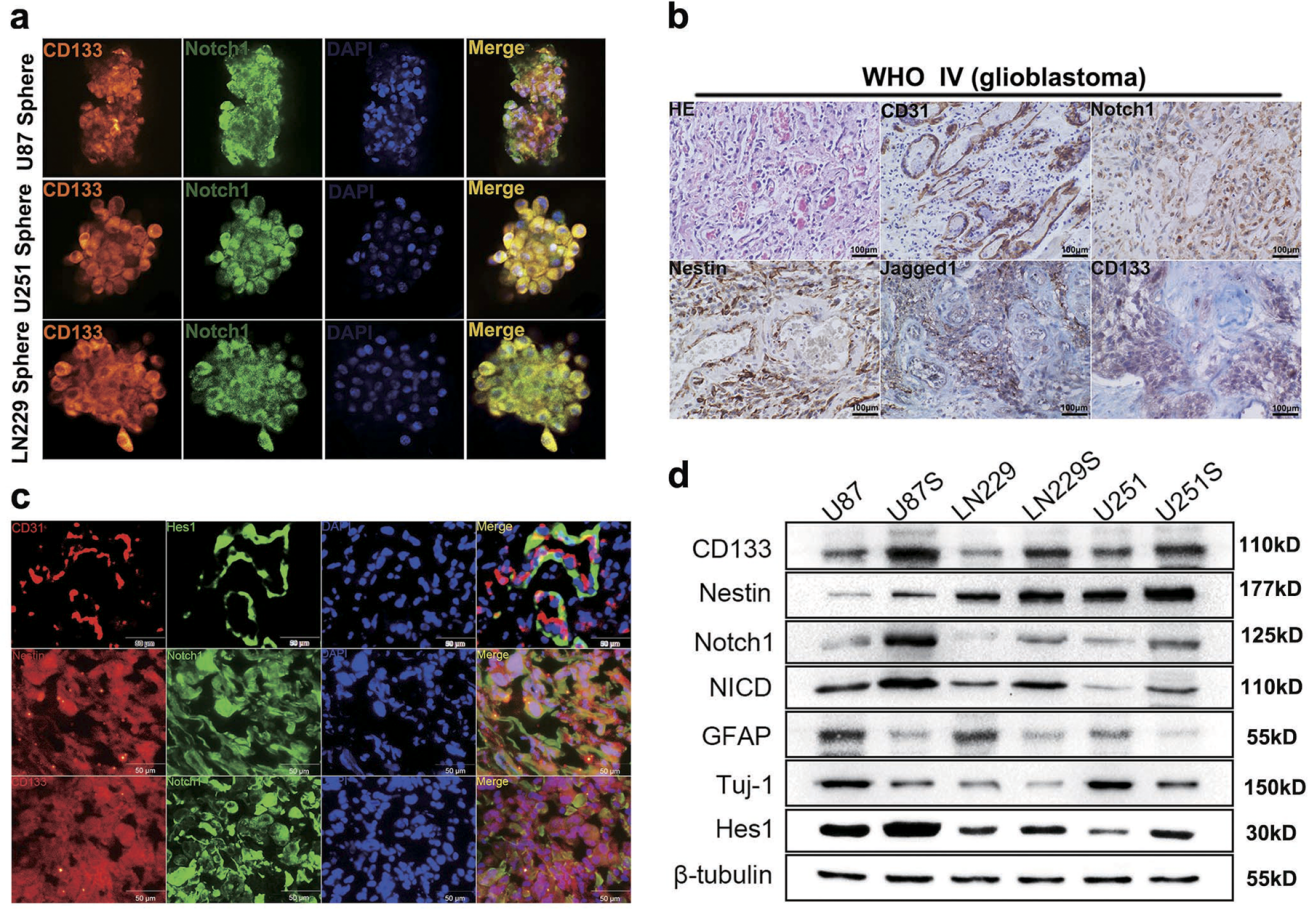
Notch1 expression was associated with stemness **a**, **d** CD133+ U87, LN229, and U251 human glioma cells that formed neurospheres exhibited higher Notch1 activity and higher expression levels of stem cell markers. The scale bar corresponds to 20 µm. **b** H&E staining and immunohistochemical staining of CD31, Notch1, Nestin, Jagged-1, and CD133 in GBM tissue. The scale bar corresponds to 100 µm. **c** Hes1 was expressed in tumor cells adjacent to CD31-expressing endothelial cells. Co-expression of Notch1 and Nestin, Notch1, and CD133 showing the corresponding colocalization in the same cells within the GBM tissue. The scale bar corresponds to 50 µm

An original method was introduced to stain neurosphere cells. Our approach maximally preserves the intact composition and morphology of spheres. Immunofluorescence staining and western blotting showed that CD133+ neurospheres expressed high levels of stemness markers (CD133 and Nestin) and components of the Notch1 signaling pathway (Notch1, NICD, and Hes1). However, the differentiation markers GFAP (glial fibrillary acidic protein, astrocyte marker) and TuJ1 (neuronal marker) were expressed at lower levels in CD133+ neurospheres (Figs. 2a, d). Next, we examined Notch1 and stemness marker expression in primary GBM sections using immunofluorescence staining. We found that Notch1-expressing cells colocalized with CD133-expressing cells and Nestin-expressing cells in primary GBM samples. Furthermore, the Notch1 target gene Hes1 was expressed in tumor cells adjacent to CD31-expressing endothelial cells (ECs; Fig. 2c). In addition, Notch1 and stemness markers also surrounded the ECs as indicated by immunohistochemical staining (Fig. 2b). These results suggested that CD133+ GBM showed elevated Notch1 activity and that a niche of ECs also has high Notch1 activity.

### Targeting Notch1 suppressed the growth and proliferation of glioma cells

**Fig. 3:**
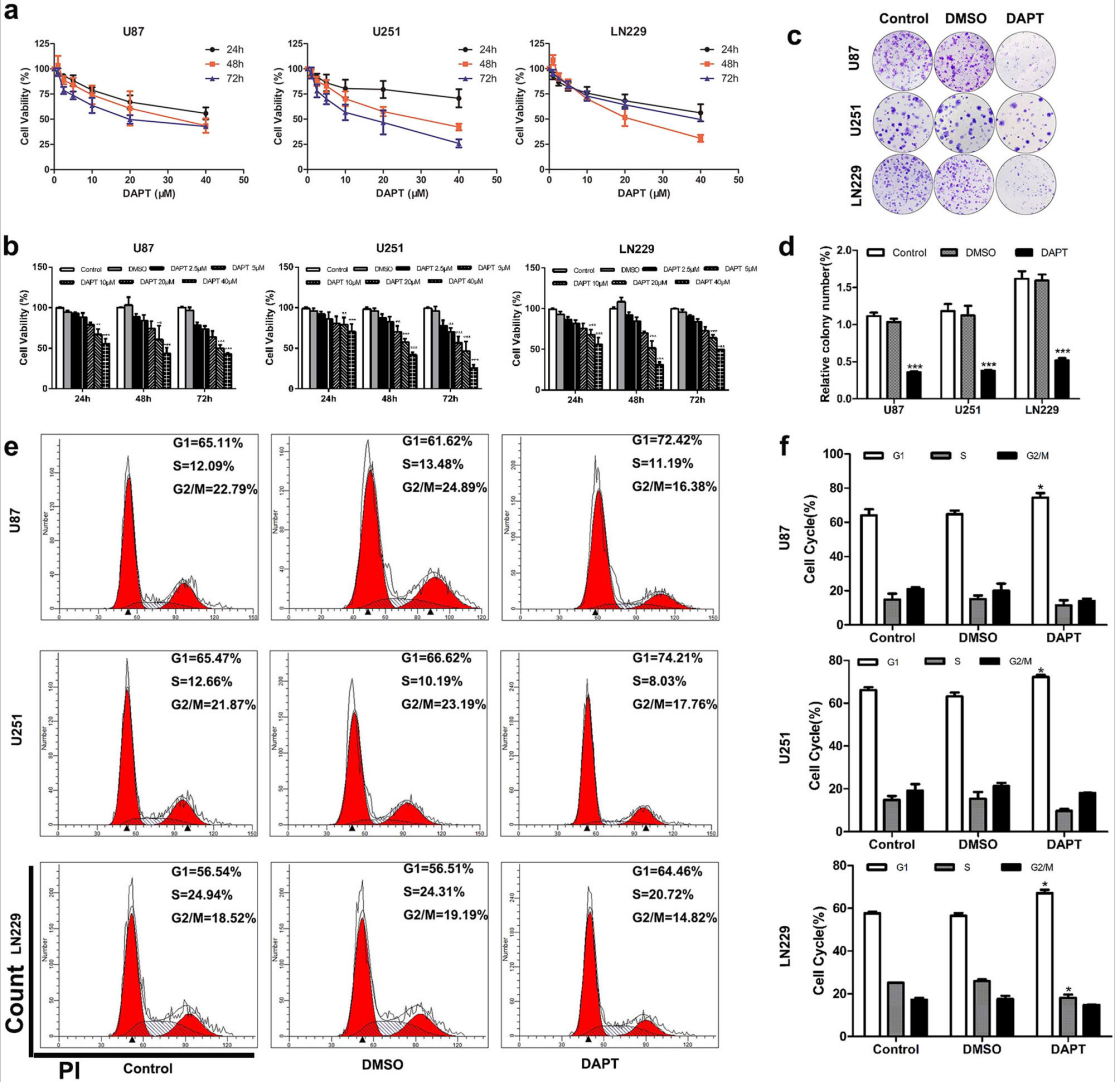
DAPT suppressed glioma cells proliferation **a, b** The effects of DAPT on U87, U251, and LN229 cells proliferation. LN229 cells proliferation was significantly impaired after DAPT treatment for 48 h as measured by the CCK8 assay. U87 and U251 cells were significantly impaired after 72-h DAPT treatment. **c, d** Reduction of colony-forming ability in DAPT-treated U87, U251, and LN229 cells. **e, f** Flow cytometry was performed to examine the G1/S arrest in U87, U251, and LN229 cells after treatment with DAPT. *P < 0.05, **P < 0.01, ***P < 0.001

U87, U251, and LN229 cells showed higher expression of Notch1 compared with A172, LN308, U118, LN18, and SNB19 cells (Fig. 1f). To study the biological function of Notch1 in GBM, we blocked Notch1 activity via chemical inhibition using DAPT (*N*-[*N*-(3,5-difluorophenacetyl)-l-alanyl]-s-phenylglycinet-butylester). The cell viability curves indicated that U251 and LN229 cells (IC_50_ = 20 μM) were more sensitive to DAPT than U87 cells (IC_50_ = 40 μM). DAPT treatment significantly inhibited U87, U251, and LN229 cells viability at 24, 48, and 72 h (*P* < 0.05, Figs. 3a, b) and the most significant reduction of cell viability occurred at 72 h (U87, U251) and 48 h (LN229) after DAPT treatment. Significant G1 phase arrest was observed in DAPT-treated glioma cells (*P* < 0.05, Figs. 3e, f). Glioma cells displayed lower colony formation efficiency after 14 days of DAPT treatment (*P* < 0.05, Figs. 3c, d). Moreover, we employed two short hairpin RNAs (shRNAs; sh1 and sh2) targeting Notch1 (Fig. 5a and Supplementary Figures S2a–d). Both shRNA1 and shRNA2 reduced Notch1 expression in glioma cells compared with control as assessed using both real-time PCR and western blotting. Colony formation and flow cytometry confirmed that Notch1 knockdown significantly restrained the proliferation of GBM cells by inducing G1 phase arrest (*P* < 0.05, Figs. 5b–d).

### Targeting Notch1 induced apoptosis of glioma cells

**Fig. 4:**
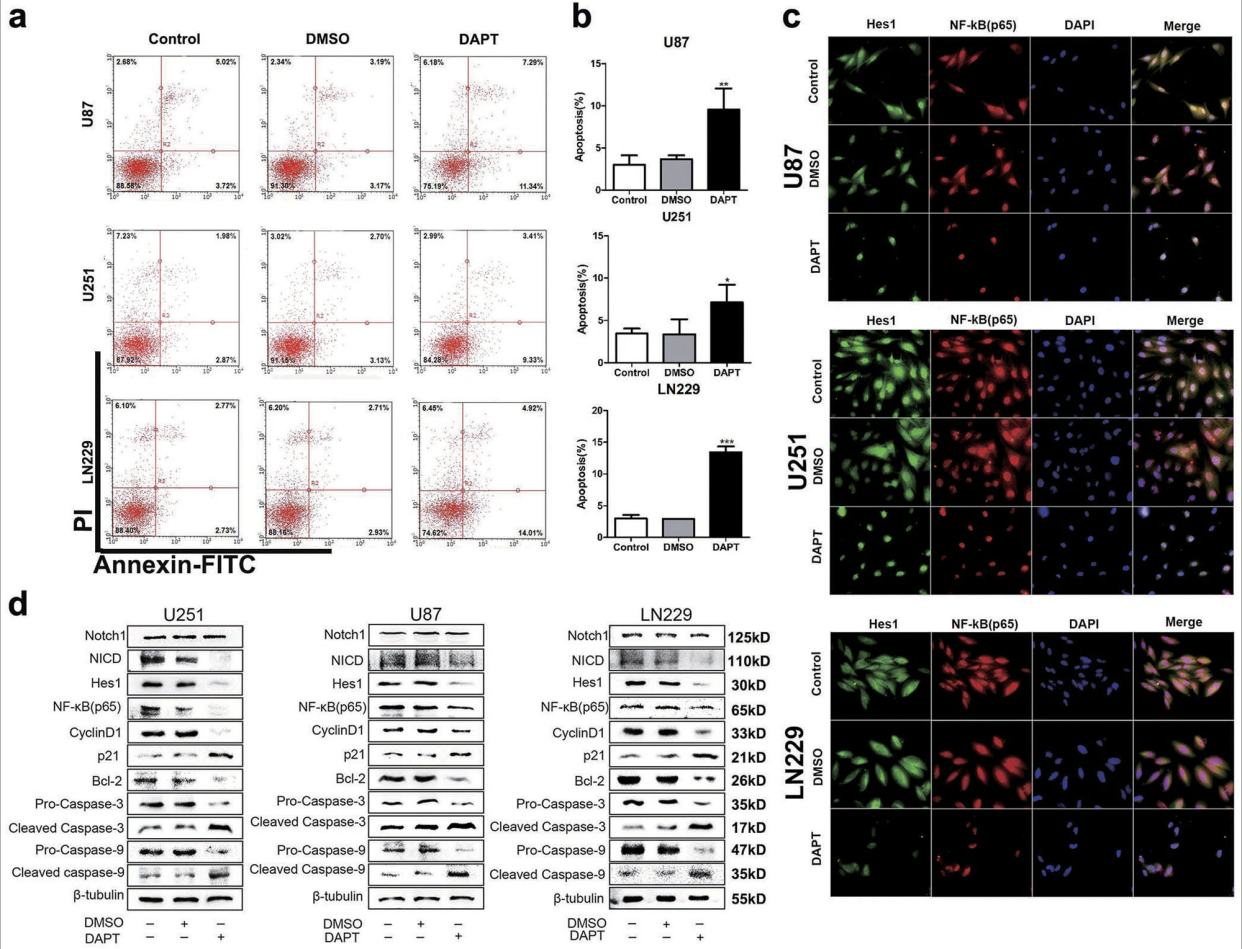
Effect of DAPT on NF-κB(p65) expression in glioma cells **a**, **b** DAPT-induced apoptosis of glioma cells in vitro. The percentages of apoptotic cells were significantly increased after DAPT treatment. **c** Immunofluorescence shows Hes1 and p65 expression in glioma cells after DAPT treatment. The scale bar corresponds to 20 µm. **d** After DAPT treatment, the Notch1, NICD, Hes1, p65, cylinD1, p21, Bcl-2, pro-caspase-3, cleaved caspase-3, pro-caspase-9 and cleaved caspase-9 expression levels were detected by western blotting. β-Tubulin was used as a loading control. *P < 0.05, **P < 0.01, ***P < 0.001

**Fig. 5:**
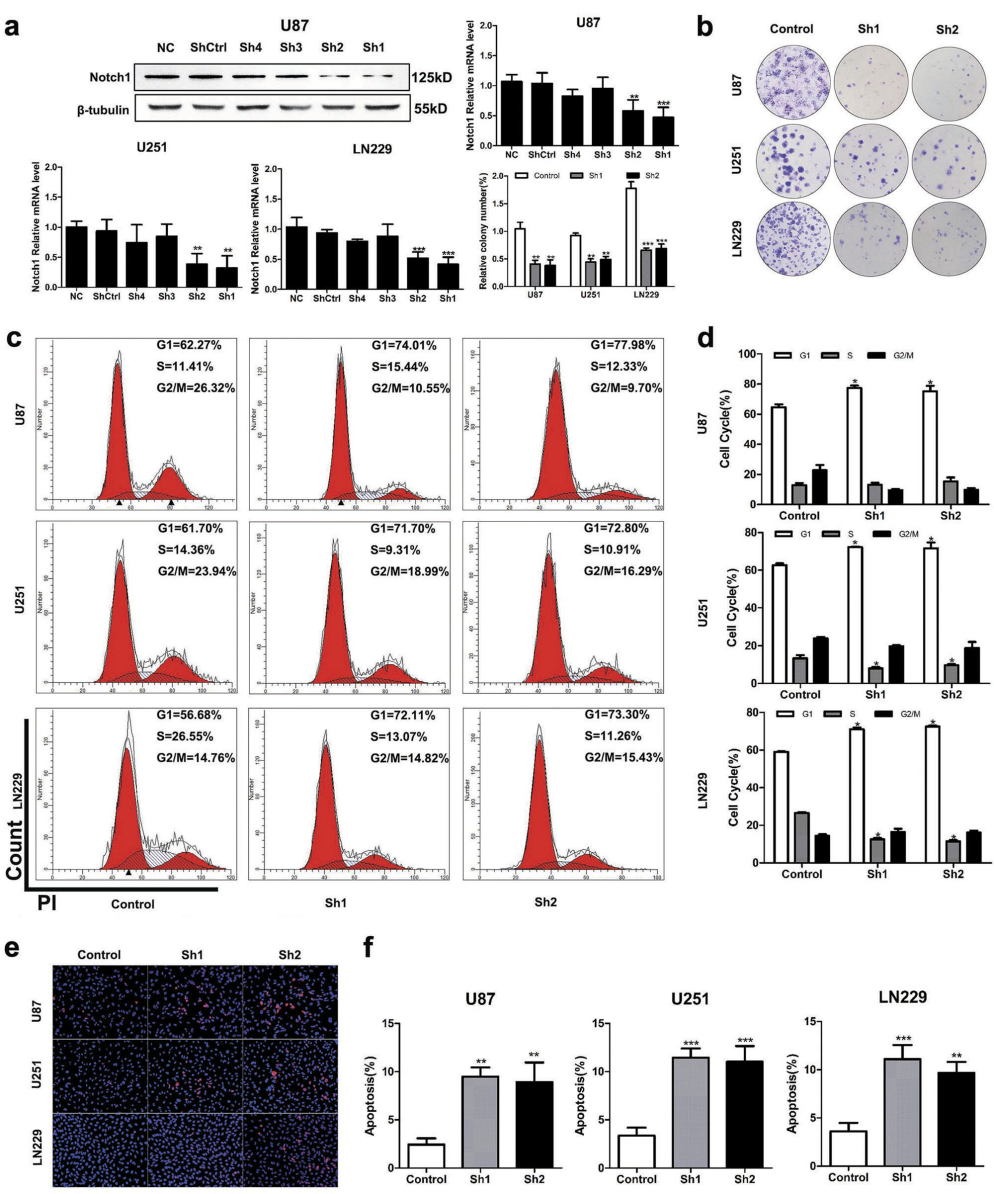
Knockdown of Notch1 suppresses proliferation and induces apoptosis in glioma cells **a** The effect of silencing Notch1 was validated by western blotting and RT-PCR. **b–d** shNotch1-transduced glioma cells were subjected to the colony formation assay and flow cytometry. **e, f** TUNEL assays were performed to examine the apoptosis of U87, U251, and LN229 cells after shNotch1 transfection *P < 0.05, **P < 0.01, ***P < 0.001

Anti-apoptosis is the common feature of cancer progression. We employed an annexin V/PI assay to evaluate the apoptotic role of DAPT in treating GBM cells. DAPT induced the early and latent phases of apoptosis in glioma cells. The number of annexin V-positive cells increased by 3.17-fold in U87 cells, 2.06-fold in U251 cells, and 4.5-fold in LN229 cells in response to DAPT (*P* < 0.05, Figs. 4a, b). Notch1 knockdown also increased Ed-dUTP labeling in glioma cells, which indicated that DNA fragmentation was induced during the apoptotic process (*P* < 0.05, Figs. 5e, f). Our results revealed that Notch1 was responsible for the excessive proliferation and reduced apoptosis of glioma cells.

### Notch1 regulated the activity of the NF-κB(p65) pathway in vitro

**Fig. 6:**
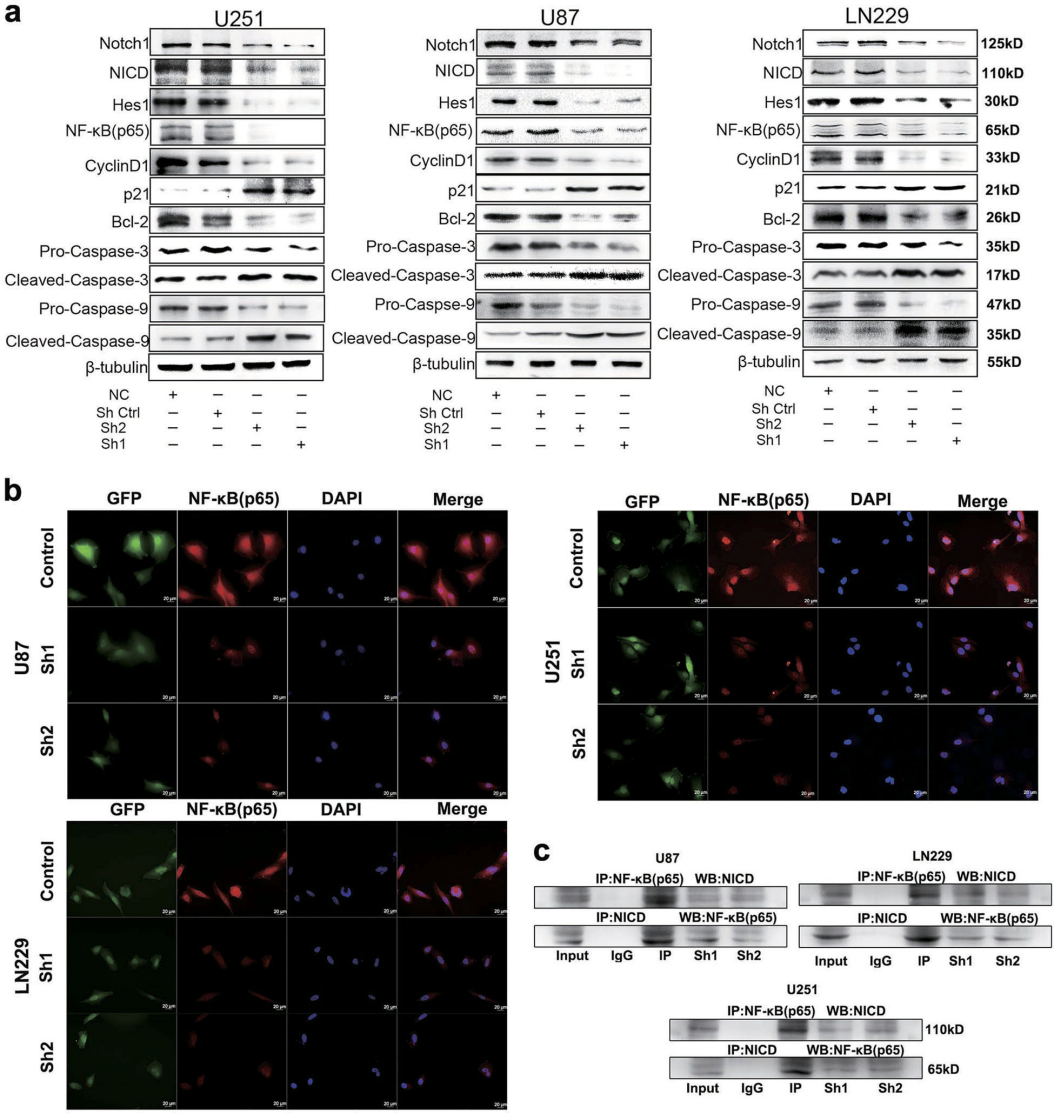
Notch1 regulates the NF-κB(p65) pathway **a** Following transfection of U87, U251, and LN229 cells with shRNA, the expression levels of Notch1, NICD, Hes1, p65, cylinD1, p21, Bcl-2, pro-caspase-3, cleaved caspase-3, pro-caspase-9, and cleaved caspase-9 were detected by western blotting. β-Tubulin was used as a loading control. **b** Immunofluorescence staining showed the distribution of NF-κB(p65) in U87, U251, and LN229 cells after shRNA treatment. **c** Three different cell lysates were denatured and then immunoprecipitated with antibodies targeting either NICD or NF-κB(p65). Both the forward and reverse immunoprecipitation showed that NICD bound to NF-κB(p65). Whole immunoglobulin (IgG) was used as a control antibody in the immunoprecipitation assays

Our previous studies suggested that Notch1 played an important role in the progression of GBM. To further investigate the underlying mechanism, we performed co-IP (co-immunoprecipitation)analysis and observed that NICD (cleaved NICD, the activated form of Notch) can bind to NF-κB(p65) (Fig. 6c). In addition, immunofluorescence staining and western blot results indicated that NF-κB(p65) was decreased after DAPT treatment and Notch1 knockdown in both cell lines (Figs. 4c, d and Figs. 6a, b). NF-κB is classically considered a pro-survival factor that induces the expression of genes regulating cell apoptosis and proliferation. Proteins regulated by NF-κB in GBM include Bcl-2 (an inhibitor of apoptosis) and cyclin D1 (facilitated tumor survival and proliferation)^17^, both of which were decreased by DAPT treatment and Notch1 knockdown (Figs. 4d, 6a).

### Knockdown of Notch1 inhibited the tumor growth activity in vivo

**Fig. 7:**
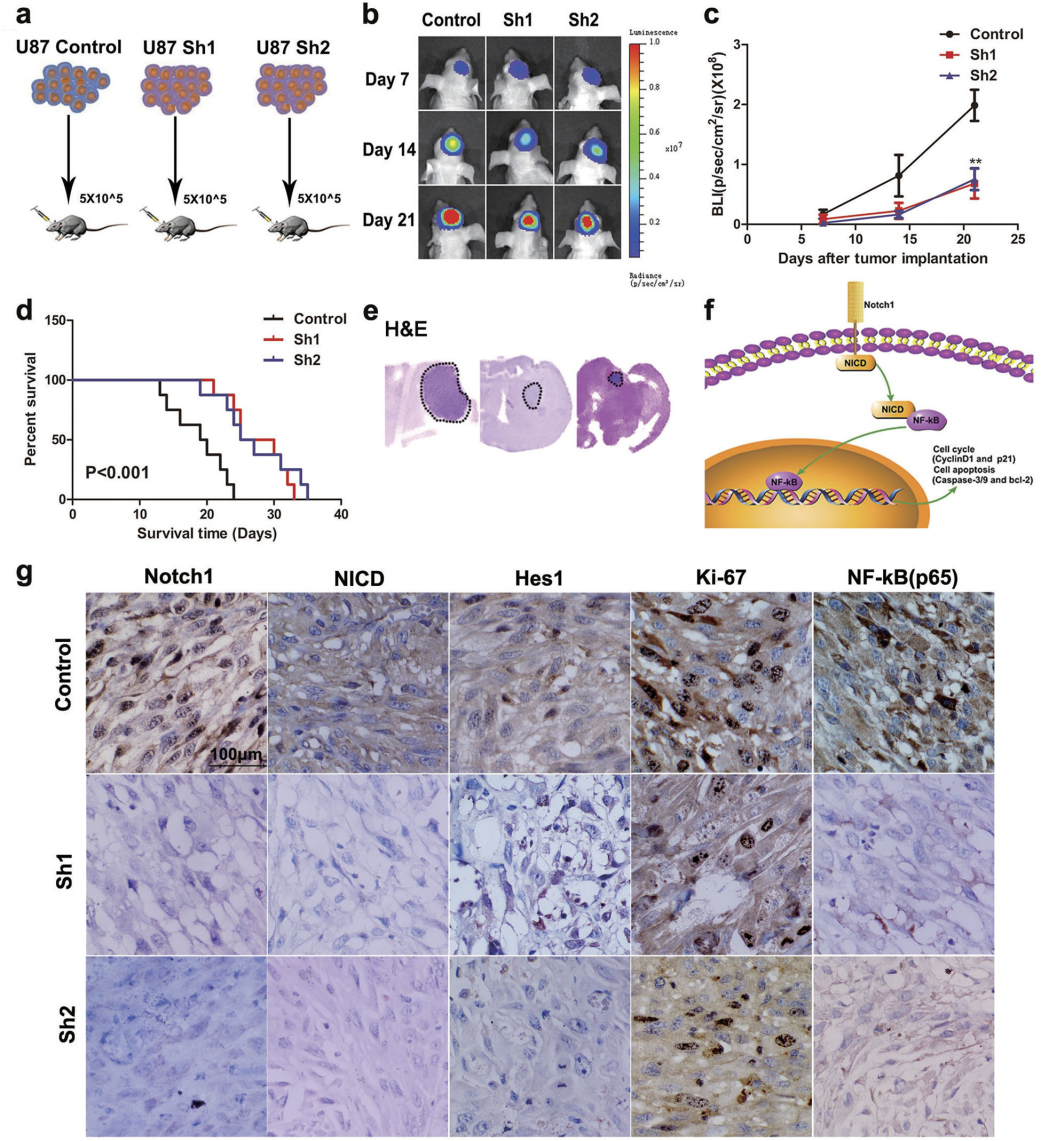
Knockdown of Notch1 inhibits U87 glioma growth in vivo **a** Flowchart of the orthotopic GBM model. **b**, **c** Bioluminescent images from the ShControl, Sh1, and Sh2 animals at 7, 14, and 21 days after tumor implantation. **d** Mouse survival in the different groups was quantified by a Kaplan–Meier curve. **e, g** H&E staining and immunohistochemistry of Notch1, NICD, Hes1, Ki-67, and NF-κB(p65) in orthotopic tumor sections. **f** Schematic mechanism of the Notch1/NICD/NF-κB(p65) signaling axis. **P < 0.01

Our in vitro study indicated that the knockdown of Notch1 can inhibit tumor cell growth. Therefore, we extended our investigation to examine whether Notch1 knockdown could produce similar effects in vivo. Then, we performed experiments according to the flowchart (Fig. 7a). After tumor implantation, bioluminescence imaging analysis of the mice revealed that tumor was stasis in the U87-Sh groups on day 21 (Figs. 7b, c). In addition, mice in the U87-Sh groups exhibited significantly longer survival times (Fig. 7d). Furthermore, IHC (Immunohistochemistry) analysis showed that the expression of Notch1, NICD, Hes1, Ki-67, and NF-κB(p65) was decreased in the U87-Sh groups, which is consistent with the in vitro results (Fig. 7g).

## Discussion

An increasing number of studies have focused on the impact of Notch1 signaling in glioma^22,23^. The expression of Notch1 in GBMs is controversial. Some articles suggest that Notch1 was overexpressed in GBMs^11,13,14^. Conversely, Espinoza et al. reported that Notch1 was absent in grade IV gliomas^12^. Notch1 may function as a tumor promoter or suppressor in different tumors^24^. To determine the role of Notch1 in GBM, we obtained 829 GBM samples from Oncomine, CGGA, and TCGA data sets. We found that the mRNA levels of Notch1 were higher in GBM than in non-neoplastic brain tissues, indicating that Notch1 acted as a tumor promoter in GBM. These findings are consistent with those from previous reports^23,25^. Notably, our findings showed that Notch1 was expressed at relatively higher levels in the classical and proneural subtypes from TCGA and CGGA databases (Fig. 1b and Supplementary Figure S1d).

Verhaak et al. reported that Notch signaling was highly expressed in the classical subtype of GBM^4^, and Norihiko et al. demonstrated that approximately 50% of proneural GBMs were positive for the Notch pathway signature^26^. To the best of our knowledge, the classical and proneural subtypes are quite different from mesenchymal and neural subtypes, which demonstrates a vast difference in biological processes^4^. Anoop et al. showed an increased prevalence of a “hybrid” state in primary GBM for two subtypes, most commonly classical and proneural (progenitor states) or mesenchymal and neural (differentiated states)^27^. These hybrid states may reflect aberrant interconversion between the phenotypic states. It has been suggested that Notch1 may play a particularly important role in GICs, a sub-population of tumor cells that have stem-like properties^21,22^. Notch inhibition induced neuronal and astrocytic differentiation^22^. We believe that Notch1 might be responsible for this dynamic transition.

GBM possesses so-called GICs, which share many NSC features such as expression of stem cell markers (i.e., Nestin, CD133), self-renewal, (i.e., continuous proliferation while maintaining an undifferentiated state), and multilineage differentiation capacity (i.e., ability to produce a heterogeneous population of differentiated cells)^28,29^. In a manner that mimics aberrant differentiation, GICs co-opt developmental programs to maintain an undifferentiated state, increasing their survival, and maintenance. The robust developmental plasticity of GICs has also been evidenced by their capacity to differentiation into ECs, γ-secretase inhibition, or Notch1 silencing blocks the differentiation of CD133+ cells into endothelial progenitors^30,31^. GICs are regulated by six main mechanisms, which include intrinsic factors such as genetics, epigenetics, and metabolism, as well as extrinsic qualities of niche factors, cellular microenvironment, and the host immune system^32^. Common pathways activated in GICs niche include Notch, BMP (Bone morphogenetic protein), NF-κB, and Wnt signaling^33–37^. Cells expressing Notch displayed higher tumor initiation capacity compared with Notch− cells, and exhibited self-renewal capacity by increasing the expression of stem cell markers such as Oct4, Sox2, and CD44^38^. Zhu et al. showed that ECs expressing Notch ligand created a stem cell niche to maintain the stem cell phenotype^39^. This observation was validated by our data showing that Notch1-expressing cells colocalized with Nestin-expressing cells and CD133-expressing cells, Hes1 is expressed in GBM cells adjacent to CD31-expressing ECs (Figs. 2a, c). The endothelial niche functions not only as a GICs niche for self-renewal but also as a prerequisite for tumor growth. We hypothesized that Notch1 could promote the survival and proliferation of GBM cells. In the present study, we showed that targeting Notch1 inhibited proliferation and induced apoptosis of GBM cells by regulating cell cycle- and apoptosis-related proteins in vitro and in vivo via suppression of the NF-κB(p65) pathway.

The Notch1 signaling pathway affects NF-κB(p65) signaling in different contexts, including GBM^18,40–42^. This was validated by our data from TCGA and CGGA (Fig. 1d and Supplementary Table S2). In cancer, NF-κB induces the transcription of genes involved in apoptosis inhibition and proliferation^43^. NF-κB regulates the transcription of cyclin D1 (an important factor for G1 progression and G1/S transition) and Bcl-2 (anti-apoptotic gene) in glioma cells^44^. In this article, the NF-κB(p65) signaling pathways are constitutively activated in glioma tissue and are also expressed at relatively higher levels in the classical and proneural subtypes in TCGA and CGGA (Fig. 1c and Supplementary Figure S1d). Pearson correlation between Notch1 and NF-κB(p65) also showed that the top score is GBM (Supplementary Table S1). This demonstrated that Notch1 and NF-κB(p65) are tightly correlated in glioma. To determine whether NF-κB(p65) was regulated by Notch1, we performed a co-IP analysis and observed that NICD can bind to NF-κB(p65) (Fig. 6c). DAPT treatment and Notch1 knockdown led to downregulation of NF-κB(p65), cyclin D1, and Bcl-2, as well as activation of p21, pro-caspase-3, and pro-caspase-9 (Figs. 4d, 6a). NICD contains at least two nuclear localization sequences on both sides of ankyrin repeats. The six ankyrin/cdc 10 repeats could be the site for protein–eprotein interaction. NICD was found to interact and activate NF-κB by competing with IκBα resulting in nuclear retention of NF-κB in T cells^45^. By analogy with IκB, the interaction of NICD with NF-κB might be through the ankyrin repeats of NICD^46–48^. Furthermore, Garner et al. used chromatin immunoprecipitation (ChIP) assays and showed that the NF-κB(p65) binds to adjacent sites in the Notch1 promoter in glioma CSCs^20^. The Notch1 pathway and NF-κB(p65) interact in a reciprocal regulatory loop in GBM cells, and this axis plays an important role in GBM carcinogenesis.

Given the central role of Notch1 signaling in glioma cells, Notch1-antagonizing strategies hold great promise in therapies of GBM. At present, gamma-secretase inhibitors (GSIs) are the most extensively explored treatments for GBM. GSIs block the terminal cleavage of NICD and thereby prevent Notch1 signaling^49^. The completed clinical trials of GSIs in GBM are a phase I trial comprising 103 patients with advanced solid tumors conducted with GSI MK-0742^50^, a phase I study of GSI RO4929097 in combination with TMZ (Temozolomide) and radiation therapy in patients with newly diagnosed GBM or World Health Organization (WHO) grade III AA^51^ and a phase I study of GSI RO4929097 with bevacizumab in patients with recurrent malignant glioma^52^. Available published data from these clinical trials have indicated that GSIs can cross the blood–brain barrier, modulate targets in the brain, and acquire a complete response in some cases of malignant gliomas^52^. Targeting Notch1 has some therapeutic effects against GBM. However, tumor recurrence could not be avoided. Identifying patients who will benefit from Notch1 inhibitors and implementing combined targeting of the Notch pathway with other pathways will likely achieve better results in clinical trials.

In this study, our results provide some novel therapeutic strategies for inhibiting the Notch1 pathway in GBM. The expression levels of Notch1 and NF-κB(p65) were prominently upregulated in proneural and classical GBM compared with the two other subtypes (neural and mesenchymal). Therefore, it might be possible that targeting Notch1 and NF-κB(p65) is more promising for treating proneural or classical GBMs rather than the other subtypes. Notch1 signaling cross-talk with NF-κB(p65) contributes to the proliferation and apoptosis of GBM. Combination drug regimens designed to prevent activity of the Notch1 signaling and NF-κB(p65) pathways may be advantageous in treating GBM.

## Materials and methods

### Cell culture

The human glioma cells U87, LN229, U251, A172, LN308, U118, LN18, and SNB19 were obtained from the China Academia Sinica Cell Repository (Shanghai, China). The cells were cultured in Dulbecco’s modified Eagle’s medium (DMEM; Gibco, Invitrogen Inc., Carlsbad, CA, USA) supplemented with 10% fetal bovine serum (Gibco) and incubated at 37 °C in 5% CO_2_. CD133+ glioma cells were collected using a CD133 MicroBead Kit (Miltenyi, GmbH, Bergisch Gladbach, Germany) following the manufacturer’s protocol. Afterwards, MACS, U87, LN229, and U251 CD133+ cells were cultured as GBM neurospheres in stem cell medium (DMEM/F12 medium supplemented with 10 ng/ml EGF (epidermal growth factor), 10 ng/ml bFGF (basic fibroblast growth factor), and B27 (1:50,Gibco)).

### Sample collection

We included all 829 available samples from three large gene expression profiling glioma cohorts. There were 128 GBM samples from the CGGA (http://www.cgcg.org.cn/) and 540 samples of GBM from TCGA (https://tcgadata.nci.nih.gov). Murat brain and Sun brain GBM samples were obtained from Oncomine (https://www.oncomine.org/). In addition, 120 glioma tumor samples and 6 non-neoplastic normal brain tissues were obtained from the Department of Neurosurgery at Tianjin Medical University General Hospital (Supplementary Table S1). All the samples were histologically graded according to the 2007 WHO Classification of Nervous System Tumors. Written informed consent was obtained from all donors and their relatives. The study was carried out in accordance with the principles of the Helsinki Declaration and approved by the ethical committee at Tianjin Medical University General Hospital.

### Tumor cell proliferation assay (CCK8 assay)

U87, LN229, and U251 cells (2 × 10^3^ cells per well) were seeded into 96-well plates. After a 24, 48, and 72-h treatment by DAPT, 10 μL of Cell Counting Kit-8 (Dojindo Laboratories, Kumamoto, Japan) was added to each well and incubated for 2 h at 37 °C. The absorbance at 450 nm was measured on a Synergy 2 microplate reader (BioTek).

### Drug treatments and lentiviral infection

U87, LN229, and U251 cells were treated with the γ-secretase inhibitor DAPT (*N*-[*N*-(3,5-difluorophenacetyl)-l-alanyl]-s-phenylglycinet-butylester; 40 μmol/L for U87 cells, 20 μmol/L for LN229 cells, and 20 μmol/L for U251 cells) (Selleck Chemicals, Houston, TX, USA) dissolved in dimethylsulfoxide (Sigma-Aldrich, St. Louis, MO, USA). Lentiviruses containing two Notch1 knockdown sequences (Sh1 and Sh2; Supplementary Table S2), and a negative control sequence (ShControl) were obtained from GeneCopoeia Inc. (USA). Lentiviral transfection was performed according to the manufacturer’s instructions as previously described^53^.

### Colony formation assay

Cells (5000) were seeded into 100-mm dish and allowed to grow for 14 days. The cells were then fixed and stained with crystal violet. The colony-forming efficiency (CFE %) was defined as the ratio of the number of colonies formed in culture to the number of cells inoculated.

### TUNEL assay

The TUNEL (TdT-mediated dUTP Nick-End Labeling) assay was performed according to the manufacturer’s instructions (Cell-Light™ EdUTP TUNEL Cell Detection Kit (Ribobio, Guangzhou, Guangdong, China)). After TUNEL staining, DAPI (Sigma-Aldrich) was used to stain the nuclei. The stained cells were imaged using fluorescence microscopy (IX73, Olympus, Tokyo, Japan).

### Apoptosis assay and cell cycle analysis

Cells were stained with annexin V/PI. The staining procedure was conducted with an Annexin V-FITC Apoptosis Detection Kit (KeyGEN, Nanjing, Jiangsu, China) according to the manufacturer’s protocol. A Bioscience FACScan Flow Cytometry System (BD Biosciences, Franklin Lake, NJ, USA) was used to detect apoptotic cells. In the cell cycle analysis, cells were fixed with 70% ethanol and incubated with RNase A (KeyGEN), after which they were stained with propidium iodide. DNA content was analyzed by flow cytometry, and the results are presented as the percentage of cells in each phase.

### Immunofluorescence

Immunofluorescence was performed in a glioma cell line and in primary GBM tumor samples. Before the cells were fixed with 4% paraformaldehyde, they were plated on glass cover slips. Tissue sections (8 μm) were sliced on a cryostat (Leica Microsystems LM3050S) and then mounted on poly-l-lysine-coated slides. Cells and tissue sections were permeabilized with 0.2% Triton-X-100 for 15 min at room temperature, blocked with 5% bovine serum albumin in phosphate-buffered saline for 20 min at room temperature, and incubated with primary antibodies at a 1:100 dilution overnight at 4 °C. Alexa fluor-labeled anti-rabbit or anti-mouse antibodies (Invitrogen, 1:500) were added to the samples. The nuclei were stained with DAPI (Sigma-Aldrich).

### Immunohistochemistry

Immunostaining was performed on paraffin-embedded sections using the avidin–biotin complex method. In brief, sections were incubated with primary antibodies (1:100 dilutions) overnight at 4 °C followed by the addition of the appropriate biotinylated secondary antibody (1:100 dilutions) (Zhongshan Biotechnology, Beijing, China) for 60 min at 37 °C. Sections were then incubated with ABC-peroxidase and diaminobenzidine (Zhongshan Biotechnology). The labeling index is presented as the percentage of positive cells among the total cell number. The slides were analyzed using NIH ImageJ software.

### Western blot and RT-PCR analysis

Western blot and real-time PCR (RT-PCR) analyses were carried out according to the manufacturer’s instructions as previously described^54^. The primary antibodies used in this study targeted the following proteins: Notch1, Hes1, Nestin, Tuj-1, CD133, and GFAP (Abcam, USA; dilution 1:1000); and NICD, NF-κB(p65), cyclin D1, p21, Bcl-2, pro-caspase-3, cleaved caspase-3, pro-caspase-9 and cleaved caspase-9 antibodies (Cell Signaling Technology (CST), USA; dilution 1:1000). β-Tubulin expression (CST; dilution 1:2000) was used as a loading control to normalize the results. For primers for Notch1 and GAPDH, see Supplementary Table S3.

### Co-immunoprecipitation

Co-immunoprecipitation assay was carried out as previously described^55^. Cells were lysed in IP lysis buffer (Thermo Fisher Scientific, Rockford, IL, USA). The cell lysates were then subjected to immunoprecipitation with either primary antibody or control immunoglobulin (Santa Cruz, CA, USA). The lysates were incubated with Protein A/G PLUS-Agarose (Thermo Fisher Scientific) overnight at 4 °C with constant agitation. The beads were then washed with wash buffer, suspended in sample buffer, and boiled, and the eluted proteins were assessed using western blotting.

### Nude mouse intracranial model

A total of 5 × 10^4^ cells infected with ShControl, Sh1, and Sh2 were intracranially injected into 4-week-old BALB/c-A nude mice (Animal Center of the Cancer Institute at the Chinese Academy of Medical Science). Bioluminescence imaging was used to detect intracranial tumor growth on days 7, 14, and 21. Body weight and overall survival were monitored. Animal experiments were approved by the Ethical Committee at Tianjin Medical University General Hospital.

### Statistical analysis

Statistical analysis was performed using SPSS 16.0. All experimental data are presented as the means ± SD of three independent experiments (IBM SPSS, Chicago, IL, USA). One-way analysis of variance was performed for comparisons among the different groups. A circus plot was achieved with the circlize package of R. *P* < 0.05 was considered statistically significant.

## Electronic supplementary material

Supplementary Table

Supplement Figure S1

Supplement Figure S2

Supplementary information
