# Supplementary material for: Notch1 is a prognostic factor that is distinctly activated in the classical and proneural subtype of glioblastoma and that promotes glioma cell survival via the NF-κB(p65) pathway
Source: Cell Death Dis. 2018 Feb 6;9(2):158. doi: 10.1038/s41419-017-0119-z (PMC5833555; doi:10.1038/s41419-017-0119-z)
Supplement: Supplementary file 1 — Supplementary Table [file 41419_2017_119_MOESM1_ESM.doc]

| Gender | | | |
| --- | --- | --- | --- |
| Male | 16 | 14 | 22 |
| Female | 24 | 26 | 18 |
| Age (Year, Mean ± SD) | 41±11.7 | 55±10.4 | 57±10.8 |
| Predominant side | | | |
| Left | 20 | 24 | 21 |
| Right | 19 | 15 | 16 |
| Middle | 1 | 1 | 3 |
| Predominant location | | | |
| Frontal lobe | 29 | 27 | 23 |
| Temporal lobe | 5 | 9 | 13 |
| Parietal lobe | 1 | 3 | 2 |
| Occipital lobe | 2 | 1 | 1 |
| Pineal body | 0 | 0 | 0 |
| Saddle area | 0 | 0 | 0 |
| Thalamus | 0 | 0 | 0 |
| Cerebellum | 3 | 0 | 1 |
| Third ventricle | 0 | 0 | 0 |

**Supplementary Table S1: The clinical features of the glioma specimens used in this study**

**Feature WHO Grade**

II(***n=*40**) III(***n=*40**) IV(***n=40***)

Abbreviation: SD, Standard deviation.

**Supplementary Table S2:**

| **NO.** | **Length** | **Target Seq(5' to 3')** |
| --- | --- | --- |
| NOTCH1-ShRNA1 | 21 | GCATGGTGCCGAACCAATACA |
| NOTCH1-ShRNA2 | 21 | GGAGCATGTGTAACATCAACA |

**Supplementary Table S3:**

The PCR primers for NOTCH1and GADPH

|  | Up stream | Down stream |
| --- | --- | --- |
| NOTCH1 | 5′-GCTACAACTGCGTGTGTGTC-3′ | 5′-GTTGGTGTCGCAGTTGGAGC-3′ |
| GAPDH | 5′-TCACTGGCATGGCCTTCCGT-3′ | 5′- CTTACTCCTTGGAGGCCAT-3′ |

**Supplementary Table S4:**

**Overview of co-expression patterns between NOTCH1 and RELA in TCGA Pan-Cancer**

Diseases or Studies Sample number Pearson Score p-value

glioblastoma multiforme 171 0.5532 4.28E-15

cholangiocarcinoma 45 0.5353 0.000151

kidney clear cell carcinoma 603 0.5085 5.83E-41

diffuse large B-cell lymphoma 47 0.4897 0.000476

brain lower grade glioma 523 0.4664 1.32E-29

kidney papillary cell carcinoma 321 0.4312 5.77E-16

rectum adenocarcinoma 103 0.4189 0.0000106

esophageal carcinoma 195 0.3637 0.000000174

prostate adenocarcinoma 548 0.3589 4.2E-18

liver hepatocellular carcinoma 421 0.3574 3.94E-14

pancreatic adenocarcinoma 183 0.34 0.00000248

pheochromocytoma &paraganglioma 185 0.3275 0.00000535

kidney chromophobe 91 0.3247 0.00169

adrenocortical cancer 77 0.3078 0.00647

colon adenocarcinoma 331 0.2994 2.78E-08

testicular germ cell tumor 154 0.2956 0.000198

lung adenocarcinoma 574 0.2639 1.35E-10

stomach adenocarcinoma 450 0.263 1.48E-08

lung squamous cell carcinoma 548 0.1729 0.0000474

uterine carcinosarcoma 57 0.1724 0.2

head & neck squamous cell carcinoma 564 0.1535 0.000254

breast invasive carcinoma 1212 0.1499 0.000000158

thyroid carcinoma 571 0.1106 0.00818

ovarian serous cystadenocarcinoma 425 0.0854 0.0786

skin cutaneous melanoma 470 0.0672 0.146

uveal melanoma 79 0.0634 0.579

uterine corpus endometrioid carcinoma 204 0.0608 0.387

cervical & endocervical cancer 309 0.0311 0.586

sarcoma 264 0.0067 0.913

bladder urothelial carcinoma 426 -0.1657 0.000597

mesothelioma 87 -0.2225 0.0383

thymoma 121 -0.231 0.0108
