# Supplementary figures and images for: Notch1 is a prognostic factor that is distinctly activated in the classical and proneural subtype of glioblastoma and that promotes glioma cell survival via the NF-κB(p65) pathway
Source: Cell Death Dis. 2018 Feb 6;9(2):158. doi: 10.1038/s41419-017-0119-z (PMC5833555; doi:10.1038/s41419-017-0119-z)

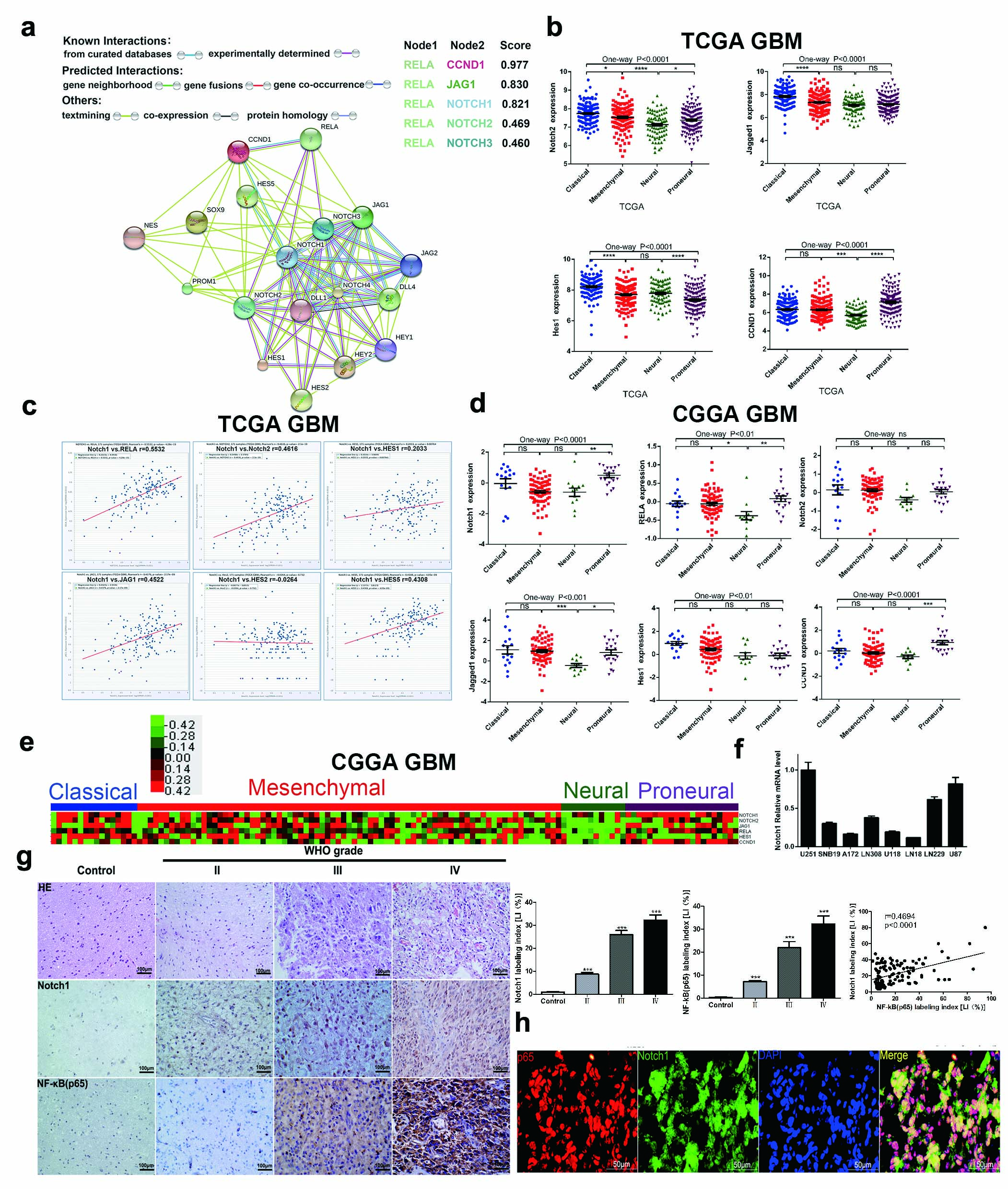

Supplement: Supplementary file 2 — Supplement Figure S1 [file 41419_2017_119_MOESM2_ESM.jpg]

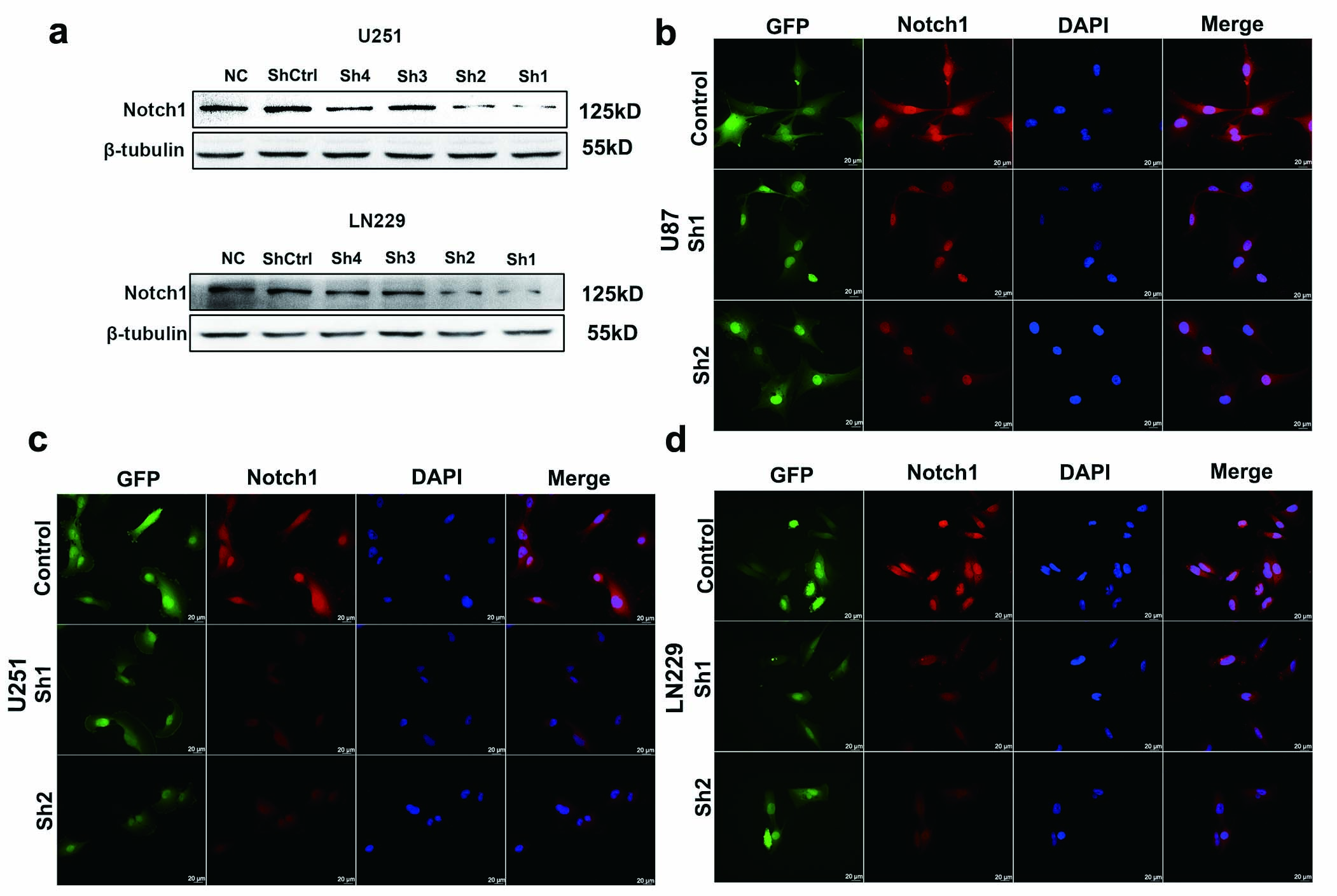

Supplement: Supplementary file 3 — Supplement Figure S2 [file 41419_2017_119_MOESM3_ESM.jpg]
