# Supplementary material for: Notch1 is a prognostic factor that is distinctly activated in the classical and proneural subtype of glioblastoma and that promotes glioma cell survival via the NF-κB(p65) pathway
Source: Cell Death Dis. 2018 Feb 6;9(2):158. doi: 10.1038/s41419-017-0119-z (PMC5833555; doi:10.1038/s41419-017-0119-z)
Supplement: Supplementary file 4 — Supplementary information [file 41419_2017_119_MOESM4_ESM.docx]

**Supplementary Figure Legends**

**Figure S1** **Correlation of the Notch1 pathway and RELA (NF-κB(p65)) in GBM. (a)** The PPI network revealed that RELA (NF-κB(p65)) is strongly associated with the Notch1 pathway. **(b)** The levels of Notch2, Jagged1, Hes1 and CCND1 were analyzed in GBM tissues from TCGA datasets. **(c)** Pearson correlation analysis between Notch1 and RELA (NF-κB(p65)), Notch2, Hes1, Jagged1, Hes2, Hes5 in TCGA datasets. **(d and e)** The levels of Notch1, RELA (NF-κB(p65)), Notch2, Jagged1, Hes1 and CCND1 were analyzed in GBM tissues from the CGGA datasets. **(f)** The mRNA levels of Notch1 in glioma cell lines were detected by RT-PCR. **(g)** Notch1 and NF-κB(p65) expression correlates with the glioma grades. Pearson correlation analysis between Notch1 and NF-κB(p65) expression. **(h)** The immunofluoresence results indicated that Notch1 and NF-κB(p65) were co-localized in the same cells of the GBM tissue. *: *P*<0.05, **: *P*<0.01, ***: *P*<0.001.

**Figure S2** **WB and immunofluorescence detected Notch1 expression in glioma cells after Notch1 knockdown treatment. (a)** Notch1 protein levels in U251 and LN229 cells infected with lentiviruses containing shRNAs targeting Notch1 were detected by Western blotting. β-Tubulin was used as a loading control. (**b, c and d)** Immunofluorescence staining shows the distribution of Notch1 in U87, U251 and LN229 cells after shRNA treatment.
